# Longitudinal trends in the association of metabolic syndrome with 550 k single-nucleotide polymorphisms in the Framingham Heart Study

**DOI:** 10.1186/1753-6561-3-s7-s116

**Published:** 2009-12-15

**Authors:** Yong-Moon Park, Michael A Province, Xiaoyi Gao, Mary Feitosa, Jun Wu, Duanduan Ma, DC Rao, Aldi T Kraja

**Affiliations:** 1 GEMS Training Program, Washington University School of Medicine, 660 South Euclid, St. Louis, Missouri 63110, USA; 2 Department of Preventive Medicine, College of Medicine, The Catholic University of Korea, Seoul, Korea; 3 Division of Statistical Genomics, Washington University School of Medicine, 4444 Forest Park Boulevard, St. Louis, Missouri 63108, USA; 4 Division of Biostatistics, Washington University School of Medicine, 660 South Euclid Avenue, St. Louis, Missouri 63110, USA

## Abstract

We investigated the association of metabolic syndrome (MetS) with a 500 k and a 50 k single-nucleotide polymorphism (SNP) gene chip in the Framingham Heart Study. We cross-sectionally evaluated the MetS longitudinal trends. Data analyzed were from the Offspring Cohort (four exams: first (*n *= 2,441), third (*n *= 2,185), fifth (*n *= 2,308), and seventh (*n *= 2,328)) and the Generation 3 Cohort (one exam: the first exam (*n *= 3,997)). The prevalence of MetS was determined using the National Cholesterol Education Program Adult Treatment Panel III diagnostic criteria, modified with a newly developed correction for medication use. The association test between an SNP and MetS was performed with a generalized estimating equations method under the additive genetic model. Multiple-testing corrections were also performed. The prevalence of MetS in the offspring cohort increased from one visit to the next, and reached the highest point by the seventh exam comparable with the prevalence reported for the general US population. The pattern of the MetS prevalence over time also reflected itself in the association tests, in which the highest significances were seen in the fifth and seventh exams. The association tests showed that SNPs within genes *PRDM16*, *CETP*, *PTHB1*, *PAPPA*, and *FBN3*, and also some SNPs not in genes were significant or close to significance at the genome-wide thresholds. These findings are important in terms of eventually identifying with the causal loci for MetS.

## Background

Metabolic syndrome (MetS) is characterized by abdominal obesity, dyslipidemia, elevated blood pressure, insulin resistance, glucose intolerance, and possibly a prothrombotic and proinflammatory state [1]. MetS is a rising global public health problem because of its role in increasing the risk of cardiovascular disease and diabetes mellitus [2]. In addition to the lifestyle or environmental risk factors, much evidence has shown that common genetic variants predispose individuals to development of MetS [3]. Framingham Heart Study (FHS) is one of the best-known cohort studies of cardiovascular disease that has demonstrated an association between cardiovascular risk factors and cardiovascular disease [4]. In the current study we investigated the longitudinal trends in MetS association with a 500 k and 50 k single-nucleotide polymorphisms (SNPs) in the FHS datasets.

## Methods

### Sampled data and MetS definition

The FHS data comprised three generations of longitudinal measurements. The first dataset is the "Original Cohort", the second dataset is the "Offspring Cohort" and the third dataset is the "Generation 3 Cohort". We analyzed data from the Offspring Cohort and the Generation 3 Cohort. The data of Original Cohort were excluded due to missing variables related to MetS. Longitudinal analysis for the prevalence of MetS and its components was performed in each of the four exams (first (*n *= 2,441), third (*n *= 2,185), fifth (*n *= 2,308), and seventh (*n *= 2,328) exams) of the Offspring Cohort, and a cross-sectional analysis was performed in the first exam (*n *= 3,997) of the Generation 3 Cohort. The prevalence of MetS was identified using the National Cholesterol Education Program (NCEP) modified diagnostic criteria with a newly developed correction for medication use [1]. One exception was that the waist circumference was not available in the FHS data distributed through Genetic Analysis Workshop 16. Therefore, we substituted it with the body mass index (BMI) criteria for obesity. Previous publications have shown that BMI is highly correlated with the waist circumference [5]. An individual with a combination of any three or more of the following risk factors was classified as having MetS: BMI ≥ 30 kg/m^2^; triglyceride (TG) ≥ 150 mg/dl; high-density lipoprotein cholesterol (HDLC) < 40 mg/dl in men and < 50 mg/dl in women; systolic blood pressure (SBP) or diastolic blood pressure (DBP) ≥ 130/85 mm Hg or use of anti-hypertensive medications; and fasting blood glucose (GLUC) ≥ 110 mg/dl or use of anti-diabetic medications with the age of diagnosis with diabetes mellitus ≥ 40 years. The modified NCEP diagnostic criteria were applied to consider the participants medication use. Thus, TG and HDL for the subjects treated with antihyperlipidemics were corrected to new values based on the following formula: TG/(1-15.2/100), HDLC/(1+6.1/100) (mg/dl). The SBP and DBP for subjects using antihypertensives were corrected to new values based on the following formula: SBP+14.8 and DBP+10.5 (mm Hg). These formulas were based on previous research, which represent corrections of a participant's medicated traits based on the mean values of many summarized treatment clinical trials [6-8].

### Statistical analysis

The qualitative MetS variable, which took medication use into account, was used to test its association with both the genome-wide scan 500 k SNPs and the additional 50 k gene SNPs, for a total of approximately 550 k SNPs (GeneChip^® ^Human Mapping 500 k Array Set and the 50 k Human Gene Focused Panel). The genotype data were recoded based on the additive model. A logistic regression of recoded genotype on MetS, based on the generalized estimating equation statistical model to account for the familial relationships among subjects within a pedigree, was performed using PROC GENMOD of SAS v 9.1.3 applying parallel computing with Platform SAS under Linux OS. In order to address multiple testing correction issues, we applied the ***simpleM ***method, which is an effective number of independent tests approach based on principal-component analysis that takes into account linkage disequilibrium (LD) information among SNPs [9]. The inferred effective number of independent tests for the 500 k chip was 281,502. Therefore, the genome-wide threshold for declaring significance for this data was a *p*-value ≤ 1.78 × 10^-7^. For the 50 k chip, based on the same method the significance threshold was 1.29 × 10^-6^. We considered these two chip results separately because they represent independent chips and also our analyses per type of chip were performed independent of each other.

## Results

### MetS: trends in prevalence

**Figure 1:**
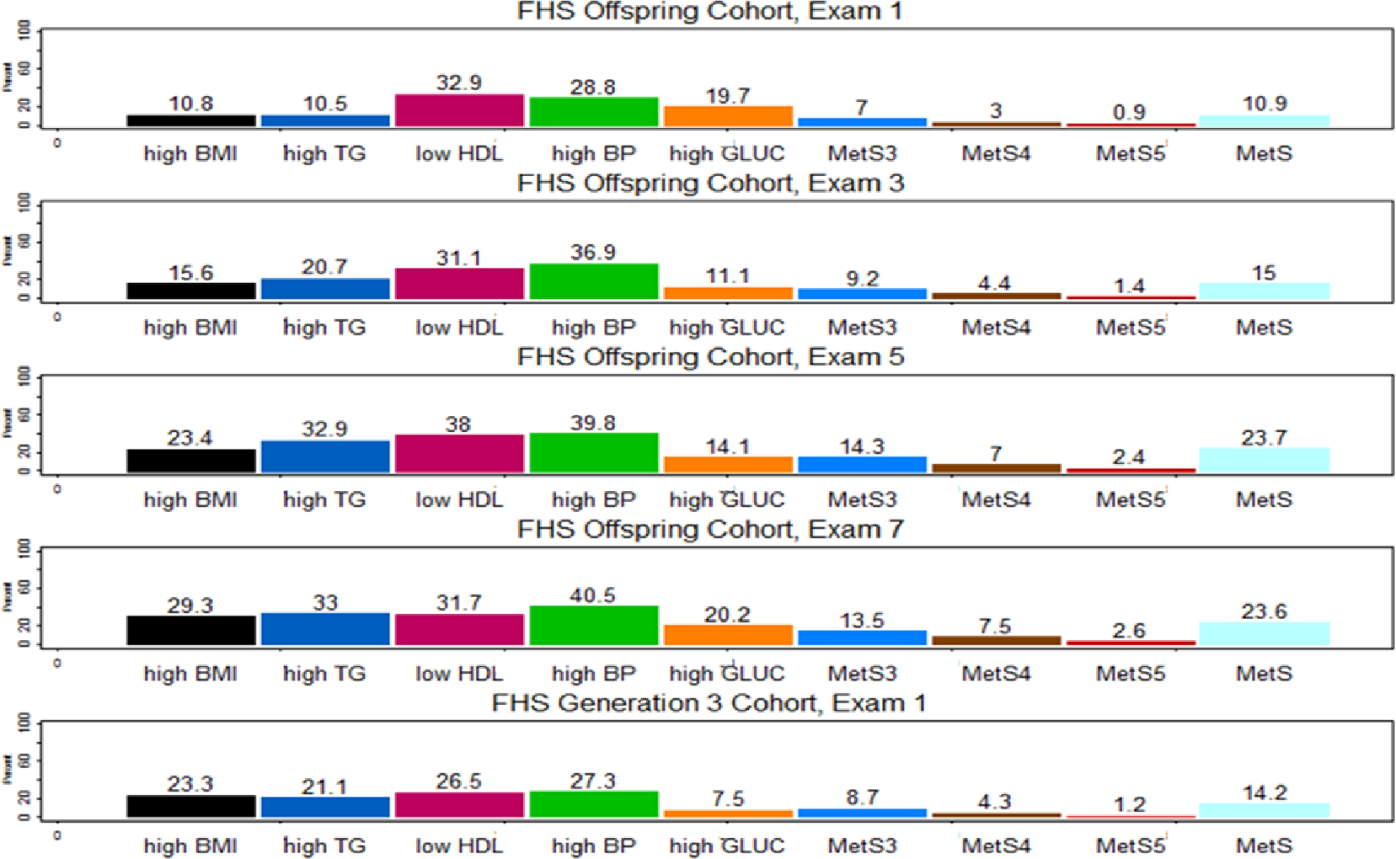
**Trends of MetS prevalence in the FHS**. The prevalence of MetS in the Offspring Cohort tended to increase with the number of exams and reached the highest point on the fifth and seventh exam, comparable with the MetS prevalence of the general US population.

The mean (range) age of subjects at the first, third, fifth, and seventh exam in the Offspring Cohort was 33.6 (9-60), 46.2 (21-72), 53.1 (28-79), and 60.1 (35-85) years, respectively. The mean (range) age of subjects in the Generation 3 Cohort was 40.2 (19-72) years. The prevalence of MetS at the first, third, fifth, and seventh exam in the offspring cohort was 10.9%, 15%, 23.7%, and 23.6%, respectively. The prevalence of MetS at first exam in the Generation 3 Cohort was 14.2%. The prevalence of subjects having four and five components of MetS beyond their threshold increased with the number of exams, while the prevalence of subjects having three components beyond the threshold decreased on the seventh exam (Figure 1). Particularly, the prevalence of high BMI, high TG, and high BP increased with exams. The prevalence of high GLUC increased with the exams, except for the GLUC at first exam. The changes in HDL prevalence had no particular trend.

### Association analysis

The association tests showed that a few SNPs within genes *PRDM16*, *CETP*, *PTHB1*, *PAPPA*, and *FBN3*, and also some SNPs not in genes, were significant or close to significance at the genome-wide thresholds for different exams in the Offspring Cohort and a few in the Generation 3 Cohort (Table 1). If the primary significant association MetS-SNP for a particular gene was from the 50 k chip, then we found that the 500 k chip for each gene reported had other SNPs that also were significant but did not reach the ultimate genome-wide thresholds. The same was observed when the primary significant association was from the 500 k. The 500 k significant SNPs findings were supported from other SNPs of the same gene on 50 k chip, but they did not pass the genome-wide thresholds. Due to space restriction, we do not report these findings.

**Table 1:** SNPs associated significantly with the qualitative MetS corrected for medication use (4 exams of offspring cohort and 1 exam of generation 3 cohort data)

						Exam 21a			Exam 23			Exam 25			Exam 27			Exam 31		
										
Marker	Chr	Position (bp)	Hugo	Role	MAF (%)	Estimate	STD error	χ^2^ *p*-value	Estimate	STD error	χ^2^ *p*-value	Estimate	STD error	χ^2^ *p*-value	Estimate	STD error	χ^2^ *p*-value	Estimate	STD error	χ^2^ *p*-value
50 k																				
rs17390167	1	3084691	PRDM16	Intron	5.2	-0.01	0.02	4.9 × 10^-1^	-0.06	0.03	**1.8 × 10^-2^**	-0.14	0.03	*3.8 × 10^-6^*	-0.04	0.03	1.4 × 10^-1^	-0.01	0.02	4.6 × 10^-1^
rs11508026	16	55556829	CETP	Intron	42.5	-0.02	0.01	1.1 × 10^-1^	-0.04	0.01	**2.0 × 10^-4^**	-0.04	0.01	**2.7 × 10^-3^**	-0.07	0.01	*7.2 × 10^-7^*	-0.01	0.01	1.6 × 10^-1^
500 k (in genes)																				
rs13241465	7	33473074	PTHB1	Intron	32.6	0.00	0.01	8.8 × 10^-1^	0.02	0.01	1.8 × 10^-1^	0.08	0.01	*1.4 × 10^-8^*	0.03	0.01	**1.3 × 10^-2^**	0.03	0.01	**1.3 × 10^-3^**
rs4236337	7	33473431	PTHB1	Intron	32.4	0.00	0.01	9.2 × 10^-1^	0.02	0.01	1.6 × 10^-1^	0.08	0.01	*2.8 × 10^-8^*	0.03	0.01	**1.9 × 10^-2^**	0.03	0.01	**2.4 × 10^-3^**
rs4509212	7	33473170	PTHB1	Intron	31.4	0.00	0.01	9.4 × 10^-1^	0.02	0.01	7.2 × 10^-2^	0.07	0.01	*6.2 × 10^-8^*	0.03	0.01	**1.5 × 10^-2^**	0.02	0.01	**1.3 × 10^-2^**
rs2418441	9	118114658	PAPPA	Intron	1.0	-0.02	0.04	6.7 × 10^-1^	0.01	0.05	8.4 × 10^-1^	0.06	0.06	3.3 × 10^-1^	-0.03	0.06	5.9 × 10^-1^	-0.25	0.04	*1.2 × 10^-8^*
rs10408896	19	8124201	FBN3	Promoter	0.1	0.03	0.14	8.3 × 10^-1^	0.41	0.16	**1.0 × 10^-2^**	0.12	0.19	5.4 × 10^-1^	0.12	0.19	5.4 × 10^-1^	0.49	0.09	*8.8 × 10^-8^*
500 k (not in genes)																				
rs318256	8				8.2	-0.01	0.02	4.6 × 10^-1^	0.00	0.02	9.2 × 10^-1^	0.02	0.02	4.4 × 10^-1^	0.01	0.02	7.2 × 10^-1^	0.08	0.01	*1.5 × 10^-7^*
rs1431573	11	126981969			43.9	-0.01	0.01	5.5 × 10^-1^	-0.06	0.01	*1.4 × 10-^7c^*	-0.03	0.01	**7.8 × 10^-3^**	-0.02	0.01	1.9 × 10^-1^	0.00	0.01	*9.4 × 10^-1^*
rs17037068	12	104333075			1.7	0.10	0.04	**3.7 × 10^-3b^**	0.27	0.04	*5.3 × 10^-10^*	0.14	0.05	**9.1 × 10^-3^**	0.14	0.05	**4.2 × 10^-3^**	-0.03	0.03	2.9 × 10^-1^
rs12437159	14	45882211			44.6	-0.02	0.01	**6.7 × 10^-3^**	-0.04	0.01	**6.2 × 10^-5^**	-0.06	0.01	*4.3 × 10^-7^*	-0.08	0.01	*1.0 × 10^-9^*	0.00	0.01	9.1 × 10^-1^
rs12147964	14	45888772			42.5	-0.02	0.01	**9.8 × 10^-3^**	-0.04	0.01	**2.7 × 10^-4^**	-0.07	0.01	*2.7 × 10^-8^*	-0.08	0.01	*7.7 × 10^-11^*	0.00	0.01	8.6 × 10^-1^
rs2899976	14	45893237			41.8	-0.02	0.01	**1.3 × 10^-2^**	-0.04	0.01	**9.5 × 10^-4^**	-0.07	0.01	*2.0 × 10^-7^*	-0.08	0.01	*2.3 × 10^-10^*	0.00	0.01	9.1 × 10^-1^
rs11625735	14	45895075			41.8	-0.02	0.01	**1.4 × 10^-2^**	-0.04	0.01	**4.7 × 10^-4^**	-0.07	0.01	*9.4 × 10^-8^*	-0.08	0.01	*1.8 × 10^-10^*	0.00	0.01	9.0 × 10^-1^
rs2010338	14	45895631			41.8	-0.02	0.01	**8.9 × 10^-3^**	-0.04	0.01	**3.4 × 10^-4^**	-0.07	0.01	*4.2 × 10^-8^*	-0.08	0.01	*1.5 × 10^-10^*	0.00	0.01	7.4 × 10^-1^
rs2415974	14	45906862			42.0	-0.02	0.01	**7.1 × 10^-3^**	-0.04	0.01	**3.0 × 10^-4^**	-0.07	0.01	*1.2 × 10^-7^*	-0.08	0.01	*6.8 × 10^-11^*	0.00	0.01	7.8 × 10^-1^
rs8009432	14	45917176			41.7	-0.02	0.01	**1.4 × 10^-2^**	-0.04	0.01	**4.4 × 10^-4^**	-0.07	0.01	*1.2 × 10^-7^*	-0.08	0.01	*3.1 × 10^-10^*	0.00	0.01	8.6 × 10^-1^
rs10498398	14	45937239			41.1	0.02	0.01	**9.8 × 10^-3^**	0.04	0.01	**1.0 × 10^-3^**	0.07	0.01	*8.6 × 10^-8^*	0.08	0.01	*1.7 × 10^-9^*	0.00	0.01	8.4 × 10^-1^
rs8019899	14	45952022			41.3	0.02	0.01	**5.6 × 10^-3^**	0.04	0.01	**1.1 × 10^-3^**	0.07	0.01	*1.2 × 10^-7^*	0.07	0.01	*3.0 × 10^-9^*	0.00	0.01	8.1 × 10^-1^

^a^Exam 21, 2 is the Offspring Cohort, and 1 is the first exam

^b^**Bold**, *p*-values support a significance tend, but do not reach the threshold

^b^*Italic*, *p*-values that pass the significance threshold and support genome-wide significance

## Discussion

The longitudinal trends of MetS prevalence and of the association tests were evaluated by using the cross-sectional phenotypic measurements. The prevalence of MetS in the Offspring Cohort increased from one visit to the next, and reached the highest point by the seventh exam, comparable with reported MetS prevalence of the US population [2]. The prevalence of MetS was also high in the young people (Generation 3 Cohort), driven mostly by the increase in obesity. This pattern was also seen in the association results, where the highest significance values were found in analysis of the later exams (fifth and seventh). SNP rs17390167, part of the gene *PRDM16 *on chromosome 1, was close to the significance threshold of the 50 k chip. This gene recently is reported to be the principal regulator of brown adipocyte tissue formation and function [10,11]. Another interesting finding was the association of rs11508026, a SNP on the *CETP *(cholesteryl ester transfer protein) gene, with the MetS. This gene is already studied extensively for its inhibitors, because inhibition of *CETP *elevates the fraction of plasma cholesterol associated with HDL [12]. SNPs rs13241465, rs4236337, and rs4509212 are part of the *PTHB1 *gene. This gene is known also as *BBS9 *gene and recognized as one of the recent Bardet-Biedl syndrome genes. Such gene polymorphisms are hypothesized to contribute to human obesity and diabetes mellitus [13].

In the Generation 3 Cohort first exam, the prevalence of MetS was moderate and comparable with those of the first and third exams of the Offspring Cohort. As a result, only a few associations were highly significant. The SNPs rs2418441 (*PAPPA*) and rs10408896 (*FBN3*) were highly significant, but at the same time one must be cautious when considering them as candidate genes for MetS, because of the rare minor allele frequency. Despite the rarity of minor allele frequency for the above two SNPs, *PAPPA *gene, a metalloproteinase, is reported to regulate the human atherosclerotic plaque [14]; *FBN3 *(Fibrilin 3) is reported to contribute in the polycystic ovary syndrome, which is associated with obesity [15]. Several other SNPs with *p*-values close to significance from the same genes mentioned in the Results and Discussion were present in the FHS data. For space reasons we did not report any of them. Finally, we would like to draw readers attention to a number of SNPs located on chromosome 14 (rs12437159, rs12147964, rs2899976, rs11625735, rs2010338, rs2415974, rs8009432, rs10498398, and rs8019899), which although not located on genes, still were associated highly with MetS.

## Conclusion

These findings are important for identifying the role of the genes reported to be associated with MetS. As such, they need to be further investigated in conjunction with the quantitative risk factors of MetS.

## List of abbreviations used

BMI: Body mass index; DBP: Diastolic blood pressure; FHS: Framingham Heart Study; GLUC: Fasting blood glucose; HDLC: High-density lipoprotein cholesterol; LD: Linkage disequilibrium; MetS: Metabolic syndrome; NCEP: National Cholesterol Education Program; SBP: Systolic blood pressure; SNP: Single-nucleotide polymorphism; TG: Triglyceride.

## Competing interests

The authors declare that they have no competing interests.

## Authors' contributions

Y-MP, ATK, MAP, and DCR conceived of the idea of this analysis. Y-MP and ATK performed the analysis and wrote the manuscript. MAP, DCR, MF, XG, JW, and DM contributed ideas to improve the manuscript. XG performed the thresholds' p-value calculations.
